# Microencapsulation of low-passage poorly-differentiated human mucoepidermoid carcinoma cells by alginate microcapsules: in vitro profiling of angiogenesis-related molecules

**DOI:** 10.1186/s12935-017-0479-6

**Published:** 2017-11-21

**Authors:** Sen Yang, Li-Juan Guo

**Affiliations:** 1 Department of Stomatology, Suining Central Hospital, No. 127, Western Desheng Road, Suining, 629000 Sichuan China; 2 Medical Beauty Department, Suining Central Hospital, No. 127, Western Desheng Road, Suining, 629000 Sichuan China

**Keywords:** Human mucoepidermoid carcinoma, Angiogenesis, Microencapsulation, 3D culture

## Abstract

**Background:**

Human mucoepidermoid carcinoma (MEC) is regarded as the most common primary salivary malignancy. High-grade MEC has a high risk of recurrence and poor prognosis. Tumor angiogenesis, induced by poorly differentiated cancer cells of high-grade MEC, contributes to tumor growth and metastasis. Therefore, elucidating molecular mechanisms underlying the pro-angiogenic ability of poorly differentiated MEC cells is critical for the understanding of high-grade MEC progression. It is well known that three-dimensional (3D) cell culture, in contrast with conventional two-dimensional (2D) culture, provides a better approach to in vitro recapitulation of in vivo characteristics of cancer cells and their surrounding microenvironment. The purpose of this study was to model a 3D environment for in vitro gene expression profiling of key molecules in poorly differentiated MEC cells for cancer neovascularization and compared them with traditional 2D cell culture.

**Methods:**

Low-passage poorly differentiated MEC cells, derived from human patient samples of high-grade MEC, were microencapsulated in sodium alginate gel microcapsules (3D culture) and compared with cells grown in 2D culture. Cancer cell proliferation was determined by MTT assays for 1 week, and gene expression of VEGF-A, bFGF and TSP-1 was analyzed by western blotting or ELISA. The hypoxic environment in 3D versus 2D culture were assessed by western blotting or immunofluorescence for HIF1α, and the effect of hypoxia on VEGF-A gene expression in 3D cultured cancer cells was assessed by western blotting with the use of the HIF1α inhibitor, 2-methoxyestradiol (2-MeOE2).

**Results:**

When encapsulated in alginate gel microcapsules, low-passage poorly differentiated human MEC cells grew in blocks and demonstrated stronger and relatively unlimited proliferation activities. Moreover, significant differences were found in gene expression, with 3D-grown cancer cells a significant increment of VEGF-A and bFGF and a drastic reduction of TSP-1. Consistently, 3D-grown cancer cells secreted significantly more VEGF-A than 2D culture cancer cells. Furthermore, 3D-grown cancer cells showed significantly higher expression of HIF1α, a molecular indicator of hypoxia; the increased expression of VEGF-A in 3D cultured cancer cells was shown to be dependent on the HIF1α activities.

**Conclusions:**

The present work shows the effects of 3D culture model by alginate microencapsulation on the proangiogenic potentials of low-passage poorly differentiated human MEC cells. Cancer cells in this 3D system demonstrate significant intensification of key molecular processes for tumor angiogenesis. This is due to a better modeling of the hypoxic tumor microenvironment during 3D culture.

## Background

Human mucoepidermoid carcinoma (MEC) is the most common type of malignant salivary gland carcinomas (SGCs) [1]. MECs are histologically heterogeneous, including variable proportions of epidermoid, intermediate and mucinous cells, which are organized into solid or cystic patterns. Based on cellular compositions and other histopathological parameters, MECs are graded into low, intermediate and high grade [1, 2]. The tumor grade is determinant to the prognosis of MEC patients, with high-grade MECs having significantly worse survival rates and higher risk of recurrence after primary surgical resection in comparison with low-grade MECs [1, 3]. However, current curative treatments for high-grade MECs are under debate and notoriously ineffective [1, 3].

Tumor angiogenesis, an integral hallmark of cancer, has been revealed as a critical step for tumor growth and metastasis [4]. In consistent with this notion, we previously found that MECs also undergo vigorous angiogenesis possibly due to in situ proliferation of vascular endothelial cells in the three-dimensional (3D) microenvironment [5, 6]. Our results implied that MEC histological grades and stages are positively correlated with cancer neovascularization [6, 7]. Furthermore, in advanced stage and/or high-grade MECs with poor prognosis, cancer cells showed higher expression levels of inhibitors of DNA binding/differentiation protein 1 (Id-1), a key pro-angiogenic transcriptional factor, and lower expression levels of thrombospondin 1 (TSP-1), a key anti-angiogenic protein ligand [6–8]. Therefore, elucidating molecular mechanisms underlying the pro-angiogenic ability of poorly differentiated high-grade MEC cells is critical for the understanding of high-grade MEC progression.

In vitro cell culture models using patient-derived cancer cell lines allow more detailed high-throughput studies of cancer-related properties and processes, such as tumor angiogenesis [9]. This has provided valuable insights into cancer progression and cancer therapies. However, such two-dimensional (2D) culture models using established human cancer cell lines have major deficiencies, including the lack of cellular heterogeneity reflective of the original malignancy and an improper tumor microenvironment, both of which are critical for cancer development and treatment resistance [9]. The former obstacle has begun to be tackled with the emerging use of tumorigenic low-passage cancer cell lines, which can better represent the heterogeneity and complexity of the parental cancers [10]. For the later obstacle, it is well known that in contrast with conventional 2D cultures, three-dimensional (3D) cell cultures provide a better in vitro approach to recapitulate in vivo characteristics of cancer cells, such as cell–cell and cell-extracellular matrix (ECM) interactions and to mimic in vivo 3D tumor microenvironment [9, 11]. In the present study, we modeled a 3D environment with an inert and biocompatible sodium alginate hydrogel matrix for low-passage poorly-differentiated MEC cells, profiled in vitro expression of key molecules for cancer neovascularization and compared them with traditional 2D cell culture.

## Methods

### Tumor tissue specimens and primary culture with tissue explants

Fresh MEC specimens were biopsied from seven human patients with clinically proven soft palate high-grade MECs, according to the revised WHO (2005)’s classification of tumors [12], at the Department of Head and Neck Tumor Surgery, West China Hospital of Stomatology, Sichuan University. Informed written consent was obtained on all participants to include their data in this study via a research protocol approved by the Ethics Committees of West China College of Stomatology, Sichuan University (#2010074).

Immediately following surgical excision, tumor tissue specimens were transferred into the pre-cooled RPMI-1640 media containing 10% (v/v) fetal bovine serum (FBS) (HyClone, Logan, Utah, USA), 105 U/L penicillin and 0.1 g/L streptomycin. These human MEC samples were transported to the laboratory on ice and primarily cultured as our previous studies [5]. In brief, in a biosafety cabinet, fresh MEC tissues were rinsed three times with RPMI-1640 complete medium and minced with sterile eye scissors into 1–2 mm^3^ chunks. Cancer tissue chunks were implanted into the culture bottles and cultured in RPMI-1640 complete medium as tissue explants in a humidified incubator (Thermo, Heraeus BB16UV, Fremont, CA, USA) with 5% CO_2_ and at 37 °C, from which primary poorly differentiated human MEC cells were derived. Characteristics of cell emigration and growth were monitored under an Olympus CK40 inverted light microscope. Culture media was changed every 2–3 days until the cells reached 70–80% confluence. For successful primary culture, poorly-differentiated cancer cells and fibroblast-like cells grew heterogeneously in individual patches. Then, these cells were digested with 0.25% (w/v) trypsin in 0.01 M PBS, and the detached fibroblast-like cells in suspension were removed from the culture bottles 2 h later. The remaining tissue explants and cells were further cultured to 70–80% confluence. Then, differential digestion procedure was repeated two times before subculturing.

### Subcultures and establishment of low-passage poorly-differentiated human MEC cell lines

After primary culture, the cells were subcultured (1:3) using 0.25% (w/v) trypsin. Poorly differentiated human MEC cells were separated from the remaining fibroblast-like cells using differential adhesion, during which fibroblast-like cells adhered faster. Until passage five, most cells were epithelial-like, poorly-differentiated human MEC cells, and fibroblast-like cells were rarely seen. The passage 5 MEC cells were further passed (1:10) using 0.25% (w/v) trypsin and separated from fibroblast-like cells using differential adhesion. These passage 6 cells were regarded as low-passage poorly-differentiated human MEC cell lines and cryopreserved for further experiments.

### Cytochemistry and immunocytochemistry for cell line identity

Cryopreserved low-passage poorly-differentiated human MEC cells derived from three patients were recovered and further expanded (1:10) using 0.25% (w/v) trypsin until passage 9. Then, these 9th passage cancer cells were collected into single cell suspension, adjusted to 2 × 10^4^ cells/mL and seeded in 2 mL/well on poly l-lysine (PLL)-coated coverslips placed in 6-well culture plates. When the 10th passage cancer cells grew to 60% confluence, the coverslips were removed, fixed with 4% paraformaldehyde (PFA) for 30 min and rinsed with PBS three times with each 5 min.

For mucin visualization, fixed low-passage poorly-differentiated human MEC cells were chemically staining with mucicarmine stain kit (Abcam, Cambridge, UK) according to the instructions provided by the manufacturer. Moreover, fixed low-passage poorly-differentiated human MEC cells were incubated overnight at 4 °C with mouse monoclonal antibodies against cytokeratin (1:100; Santa Cruz Biotechnology, Santa Cruz, CA, USA) or mouse monoclonal antibodies against vimentin (1:100; Thermo, Fremont, CA, USA). After rinsing in PBS, cells were further incubated with biotin-conjugated goat anti-mouse secondary antibody (ZSGB-Bio, Beijing, China) and rinsed. Then, cells were incubated with streptavidin-peroxidase complexes (ZSGB-Bio) and the immunoreactive signals were developed by the DAB substrate (ZSGB-Bio). Negative controls included omission of primary or secondary antibodies and substitution of primary antibodies with isotype controls. After mounting in glycerol jelly mounting medium (Beyotime, Beijing, China), stained cells were visualized and imaged with an Olympus IX70 inverted light microscope.

### Cell microencapsulation by alginate gel microcapsules and 3D culture

Cell microencapsulation by alginate gel microcapsules was performed as previously reported [13]. In brief, cryopreserved low-passage poorly-differentiated human MEC cells derived from three patients were recovered and further expanded (1:10) using 0.25% (w/v) trypsin until passage 9. Then, those 9th passage cancer cells were collected at logarithmic phase into single cell suspension. After centrifugation, cancer cell pellets were re-suspended (4 × 10^6^ cells/mL) in 1.5% (w/v) sodium alginate (Santa Cruz Biotechnology) previously diluted in sterile complete medium containing 300 mM mannitol. The cell suspension was passed through a syringe pump mounted to the high-voltage pulse microcapsule molding apparatus (custom product of College of Chemical Engineering, Sichuan University) with pre-defined parameters (needle diameter, voltage, flow rate and distance from needle to gelling solution). In a pilot study, our parameter set enabled the elaboration of microcapsules with a mean diameter of 800 μm. The droplets were gelled in 100 mM calcium chloride (CaCl_2_) dissolved in 150 mM mannitol/ultrapure water solution for 15 min. Alginate-PLL-alginate (APA) microspheres were made with 0.05% (w/v) PLL and 0.1% (w/v) alginate. Then, hollow APA microcapsules were made by incubating solid APA microspheres in citrate buffer solution (55 mM) for 10 min. After the residual debris were washed off, low-passage poorly-differentiated human MEC cells encapsulated in APA microcapsules were cultured in sterile RPMI-1640 complete medium in a humidified incubator with 5% CO_2_ and at 37 °C.

### Scanning electron microscopy of alginate gel microcapsules

Alginate gel microcapsules with low-passage poorly-differentiated human MEC cells were placed overnight in a lyophilizer. The lyophilized microcapsules were treated with metal spray. The overall structures and the outer or inner surfaces of microcapsules were scanned by a scanning electron microscope to determine whether the walls of alginate gel microcapsules meet the requirements for a semi-permeable membrane.

### MTT assay for cell proliferation

The viability of APA microcapsule-cultured and conventional 2D MEC cells was assessed by the MTT assay. APA microcapsules with a total volume corresponding to approximately 1.2 × 10^6^ cells per well in a 24-well plate were maintained for 7 days under the same conditions mentioned above. Low-passage poorly-differentiated human MEC cells of the same batch used in APA microcapsulation were passed and maintained as 2D culture for 7 days with the initial seeding quantity the same as in 3D microcapsule culture. Three duplicates were included for MEC cells derived from one patient, and MTT assays were independently repeated thrice with MEC cells derived from three patients. MTT assay was performed as previously reported [14]. Briefly, at the designated time, MEC cells from 3D and 2D culture groups were harvested and incubated with methylthiazolyl tetrazolium (MTT) (Santa Cruz Biotechnology) solution at 37 °C for 24 h. The cells were washed three times and added DMSO to develop a purple color. The light absorbance was read with an ELISA reader (MS2353, AniLabsystem Co. Ltd, Vantaa, Finland) at 570 nm with reference at 630 nm, and optical density (OD) values were calculated for graph plotting of cancer cell growth curves.

### Western blotting

The protein expressions of key molecules in MEC cells for cancer neovascularization and hypoxia were assessed by western blotting. For the comparison between 2D and 3D culture, experimental group design and cell culture were the same as MTT assay. For the effect of hypoxia on VEGF-A gene expression during 3D culture, we applied the HIF1α inhibitor, 2-methoxyestradiol (2-MeOE2) (Selleck, S1233; Houston, TX, USA). In brief, 6 days after 3D culture, 2-MeOE2 (50 ng/μL) or the same amount of DMSO were added into the culture medium and washed off at the end of the 7th day of culture.

After 7 days in culture, total protein extracts were prepared using RIPA lysis buffer (Beyotime). The protein content was determined with BCA method (Beyotime), and equivalent amounts of total proteins (10 μg) were resolved on 10% SDS-PAGE gel. After transferring to PVDF membrane (Beyotime), protein expression levels were probed at 4 °C overnight by immunoblotting using mouse monoclonal primary antibodies specific for vascular endothelial growth factor A (VEGF-A) (Santa Cruz Biotechnology), basic fibroblast growth factor (bFGF) (Santa Cruz Biotechnology), TSP-1 (Sigma-Aldrich, St. Louis, MO), hypoxia-inducible factor 1 alpha (HIF1α) (Abcam) and β-actin (Sigma-Aldrich), respectively. Then, the membranes were incubated with peroxidase-conjugated rabbit secondary antibodies against anti-mouse IgG (Beyotime) for 1 h at 25 °C, and immunoblots were developed with the enhanced chemiluminescence (ECL) reagents (Beyotime). The blotted membranes were visualized with a ChemiDoc™ system (Bio-Rad), and chemiluminescence signals were quantified by the software Quantity One (Bio-Rad).

### Enzyme-linked immunosorbent assay (ELISA) for VEGF-A in 2D versus 3D culture medium

Seven days after 2D or 3D culture, the release of the pro-angiogenic VEGF-A from cancer cells was quantitatively examined from the respective medium samples using the human VEGF-A ELISA system (Invitrogen, BMS277-2; Waltham, MA, USA) with the recombinant VEGF-A as a standard. Samples and standards were performed in duplicate, and each group contains three independent samples.

In brief, the pre-coated microwells from the microplate were washed twice with approximately 400 μL wash buffer per well. After washing, the pre-diluted media (1:2 dilution) from 2D or 3D culture and different concentrations of standards were added to the wells. Plates were incubated for 2 h at room temperature. After an additional wash with PBST, 100 μL of biotin-conjugated anti-human VEGF-A polyclonal antibody (1:100) was added to the wells and incubated for 1 h at room temperature. After washing with PBST, 100 μL of horseradish peroxidase-labeled streptavidin (1:100) was added to the wells and incubated for 1 h at room temperature. After washing with PBST, color was developed in 3,3′,5,5′-tetramethylbenzidine liquid substrate for about 30 min at room temperature and then stopped with the stop solution. The plate was analyzed with an enzyme immunoassay (EIA) plate reader at a wavelength of 450 nm (Thermo).

### HIF1α immunofluorescent staining of 2D versus 3D cultured cancer cells

Fixed low-passage poorly-differentiated human MEC cells from 2D and 3D culture were incubated overnight at 4 °C with mouse monoclonal antibodies against HIF1α (1:200; Abcam). After rinsing in PBS, cells were further incubated with Alexa Fluor 488-conjugated goat anti-mouse secondary antibody (1:500; Abcam) and rinsed. Negative controls included omission of primary or secondary antibodies and substitution of primary antibodies with isotype controls. After mounting in anti-fading mounting medium (Beyotime, Beijing, China), stained cells were visualized and imaged with a fully motorized, Olympus IX83 inverted wide-field epifluorescence microscope with a DP80 CCD camera under the monochromatic mode controlled by Olympus cellSens Dimension software (Olympus; Tokyo, Japan).

### Statistical analysis

All quantitative data were presented as mean ± standard deviation (SD) and statistically analyzed with IBM SPSS Statistics 22.0 (IBM; Armonk, NY). For MTT assay for cell viability, the differences between 3D and 2D culture groups at serial time points were analyzed by the two-way repeated-measures analysis of variance (two-way RM-ANOVA) followed by Dunn’s post-test. For relative protein expression by western blotting or ELISA, all the comparisons between 3D culture group and 2D culture group were made by independent sample tests or one-way RM-ANOVA followed by Tukey’s post-test for multiple comparisons. For all data analysis, *P* < 0.05 was considered as statistical significance. All of the statistical graphs were plotted using GraphPad Prism 6.01 (GraphPad Software, Inc.; Northampton, MA) and assembled into the figures of this publication with Adobe Illustrator CC 2014.

## Results

**Fig. 1:**
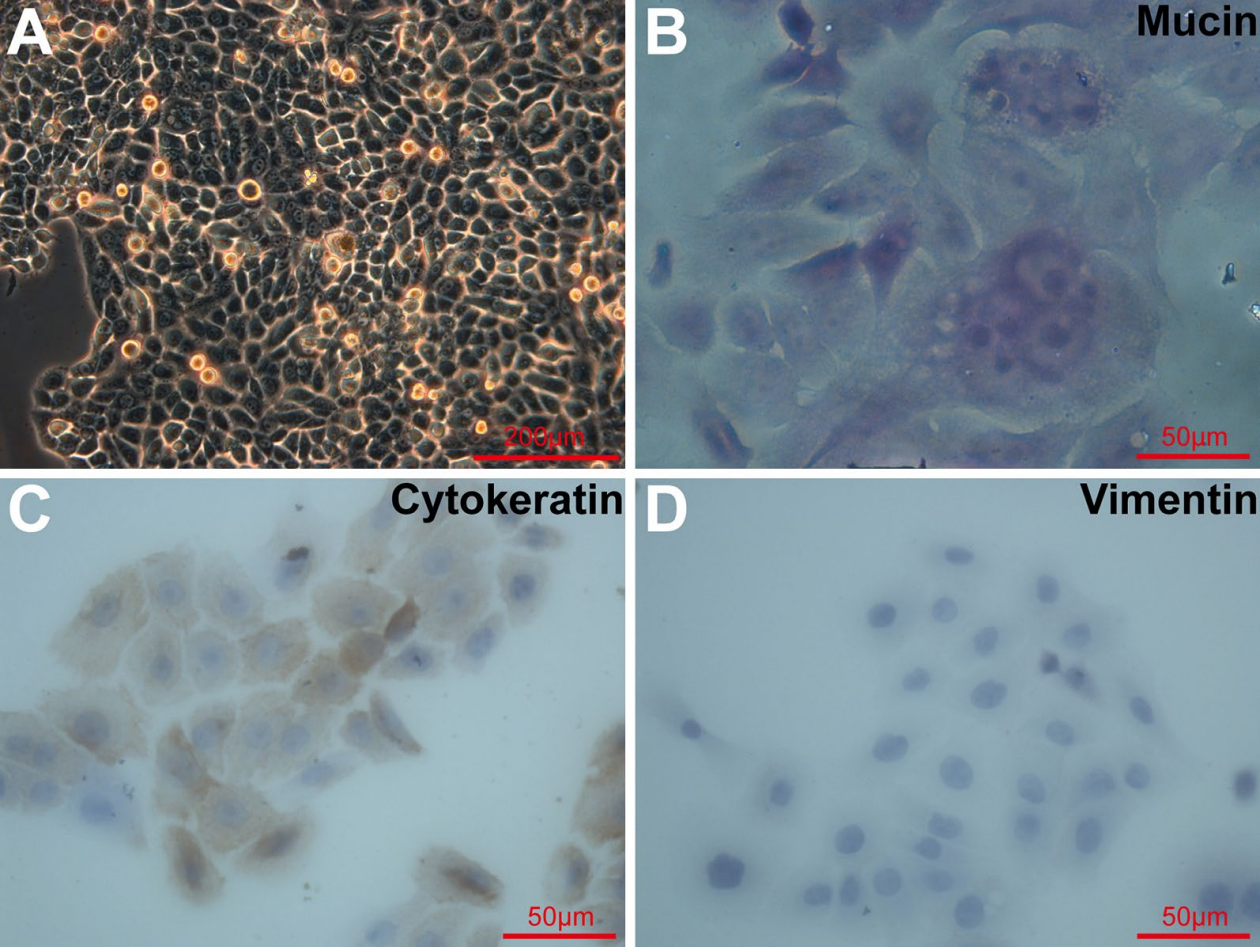
Morphological and molecular characteristics of low-passage poorly-differentiated, high-grade human MEC cells cultured in 2D. **A** Representative phase-contrast microscopic images of passage 10 poorly-differentiated human MEC cells. **B** Representative bright-field microscopic images of mucicarmine staining on passage 10 poorly-differentiated MEC cells. **C**, **D** Representative bright-field microscopic images of immunocytochemistry for cytokeratin (**C**) and vimentin (**D**) on passage 10 poorly-differentiated MEC cells. Three independent repeats, with each triplicates, were performed

When cultured in 2D, the heterogeneous low-passage poorly-differentiated human MEC cells (passage 10) were observed predominantly as polygonal epidermoid cells with variable cell sizes. Those cancer cells all exhibited a giant and rounded nucleus and showed a high karyoplasmic ratio (Fig. 1A). Some of these epidermoid cells showed mitosis (Fig. 1A). There were also some small or larger and more oval cells, presumably the intermediate cancer cells (Fig. 1A). Multinucleated giant cells or megakaryocytes were rarely observed (Fig. 1A). Moreover, some but a few of those cancer cells were revealed by mucicarmine staining as mucin-producing mucinous cells in individuals and clusters (Fig. 1B). Those mucinous cells also showed their characteristic morphology, acinar pyramidal or goblet-like (Fig. 1B). While the positive staining of cytokeratin in the cytoplasm and membrane of nearly all the MEC cells demonstrated their epithelial origin (Fig. 1C), the negative staining of vimentin of all the MEC cells exclude cells of mesenchymal origin (Fig. 1D). Those morphological and molecular characteristics were consistent with poorly-differentiated cancer cells seen in high-grade human MECs [1, 2], which implied the successful establishment and maintenance of heterogeneous low-passage poorly-differentiated human MEC cell lines.

**Fig. 2:**
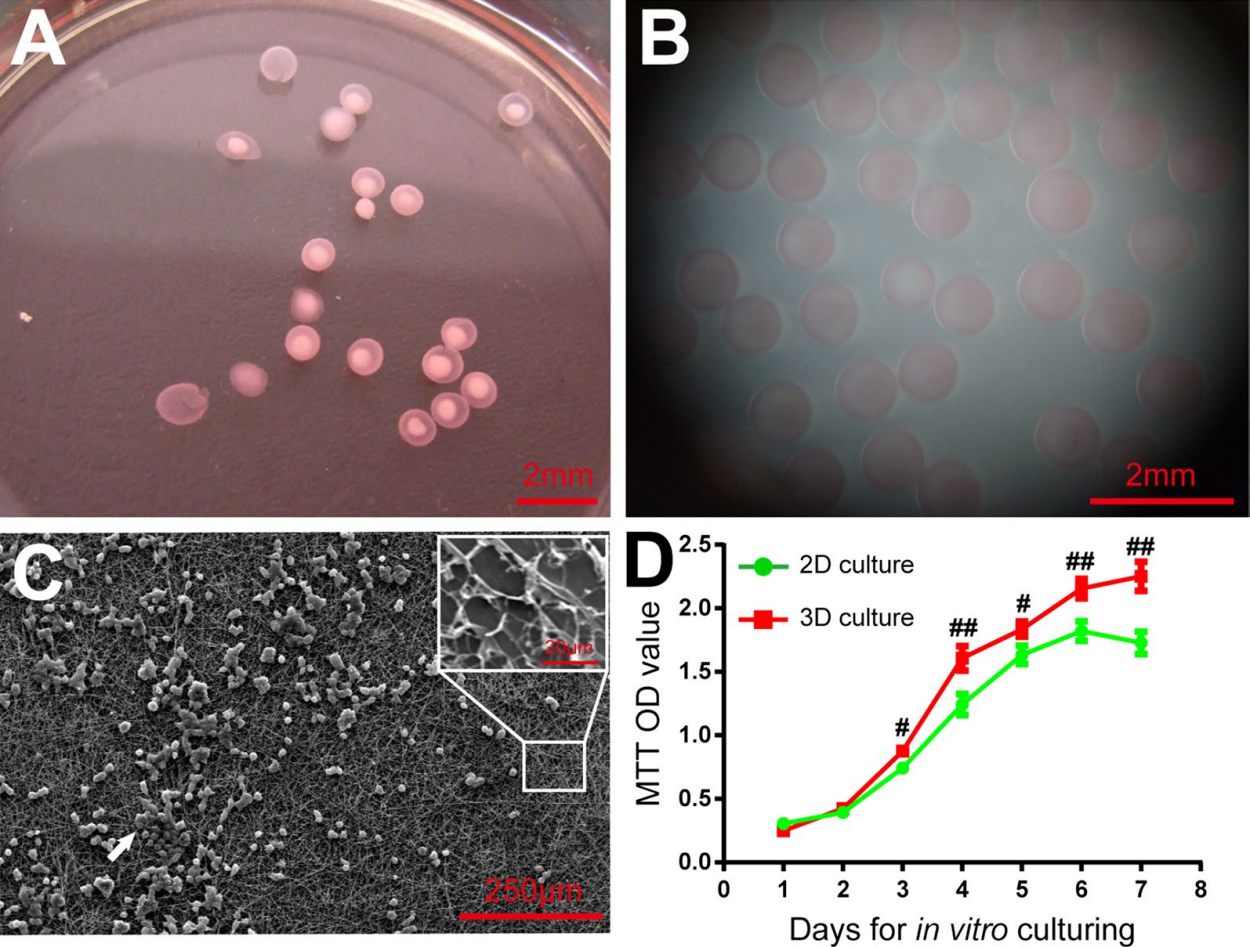
Growth characteristics of low-passage poorly-differentiated, high-grade human MEC cells cultured in 3D APA microcapsule microenvironments. **A** Representative macroscopic images of cell-loaded APA capsules 24 h after cell microencapsulation. **B** Representative stereomicroscopic images of cell-loaded APA capsules 7 days after culture. **C** Representative SEM images of the inner surfaces of cell-loaded APA capsules. **D** Cell viabilities of passage 10 poorly-differentiated human MEC cells cultured in 2D and 3D. Three independent repeats, with each triplicates, were performed. 2D vs. 3D: ^#^ *P* < 0.05; ^##^ *P* < 0.01

Low-passage poorly-differentiated human MEC cells of the same batch for 2D cell culture were encapsulated in APA microcapsules (Fig. 2A) and maintained for a period of 7 days. The cell-loaded APA microcapsules have smooth and luster surfaces (Fig. 2A, B) and remained intact without dissolution or damage after the time of culture (Fig. 2B). Microencapsulated poorly-differentiated human MEC cells grew in cell microspheres and remained within the APA microcapsules (Fig. 2B). Scanning electron microscopy showed that microencapsulated poorly-differentiated human MEC cells adhered onto the inner surfaces of the APA microcapsules, which were filled with high densities of micropores of similar sizes (Fig. 2C). Those micropores would facilitate cancer cell interactions and growth [13]. In consistent with this assumption, our results showed that in comparison with 2D cell culture, poorly-differentiated human MEC cells cultured as 3D in the APA microcapsules proliferate stronger and relatively unlimited (Fig. 2D). In detail, significantly higher proliferation activities were seen for 3D cultured cancer cells during late but not early culture periods (1st day: 2D, 0.3053 ± 0.0198; 3D, 0.2493 ± 0.0170, *P* > 0.05; 2nd day: 2D, 0.3923 ± 0.0254; 3D, 0.4240 ± 0.0127, *P* > 0.05; 3rd day: 2D, 0.7423 ± 0.0272; 3D, 0.8760 ± 0.0272, *P* < 0.05; 4th day: 2D, 1.2430 ± 0.0481; 3D, 1.6060 ± 0.0560, *P* < 0.01; 5th day: 2D, 1.6330 ± 0.0412; 3D, 1.8350 ± 0.0340, *P* < 0.05; 6th day: 2D, 1.8200 ± 0.0443; 3D, 2.1540 ± 0.0441, *P* < 0.01; 7th day: 2D, 1.7280 ± 0.0512; 3D, 2.2510 ± 0.0659, *P* < 0.01). Moreover, while cell proliferation reached the plateau after 5 days during 2D culture, there were no signs of proliferation plateau during 3D culture. These results indicated that the APA microcapsule provides a suitable model of 3D culture for low-passage poorly-differentiated human MEC cells.

**Fig. 3:**
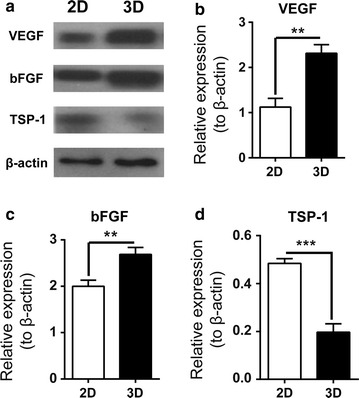
Comparative protein expression analysis of angiogenic inducers and inhibitor for low-passage poorly-differentiated human MEC cells cultured in 2D and 3D microenvironments. **a** Representative immunoblot images of key angiogenic inducers and inhibitor for passage 10 poorly-differentiated high-grade human MEC cells cultured in 2D and 3D microenvironments. **b**–**d** Quantitative comparison of VEGF (**b**), bFGF (**c**) and TSP-1 (**d**) protein expression for passage 10 poorly-differentiated MEC cells cultured in 2D and 3D. Three independent repeats, with each triplicates, were performed. 2D vs. 3D: **P < 0.01; ***P < 0.001

**Fig. 4:**
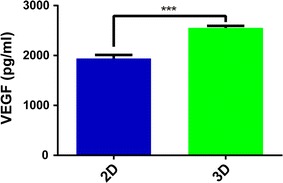
The release of the pro-angiogenic inducer VEGF-A from 2D versus 3D cultured cancer cells. Quantitative analysis with the human VEGF-A ELISA kit. Three independent repeats, with each duplicates, were performed. 2D vs. 3D: ***P < 0.001

Then, we assessed protein expression of key molecules for cancer neovascularization [4] in 3D cultured low-passage poorly differentiated MEC cells and compared them with traditional 2D cell culture. There was a significant increase for 3D cultured cancer cells in the protein expression of key pro-angiogenic molecules, such as VEGF-A (3D: 2.313 ± 0.110 vs. 2D: 1.124 ± 0.111, *P* < 0.01) (Fig. 3a, b) and bFGF (3D: 2.687 ± 0.086 vs. 2D: 1.999 ± 0.077, *P* < 0.01) (Fig. 3a, c). Furthermore, the protein expression of TSP-1, a key anti-angiogenic molecule, was drastically reduced in 3D cultured cancer cells (3D: 0.197 ± 0.020 vs. 2D: 0.484 ± 0.011, *P* < 0.001) (Fig. 3a, d). Next, we further examined with ELISA the release of these growth factors for tumor angiogenesis into the tumor microenvironments, taking VEGF-A as an example. We found that 3D cultured cancer cells secreted significantly more VEGF-A than 2D culture cancer cells (3D: 2553 ± 40.26 pg/mL vs. 2D: 1941 ± 71.07 pg/mL, *P* < 0.001) (Fig. 4). Therefore, low-passage poorly differentiated MEC cells cultured in a 3D microenvironment demonstrated significant intensification of key molecular processes for tumor angiogenesis.

**Fig. 5:**
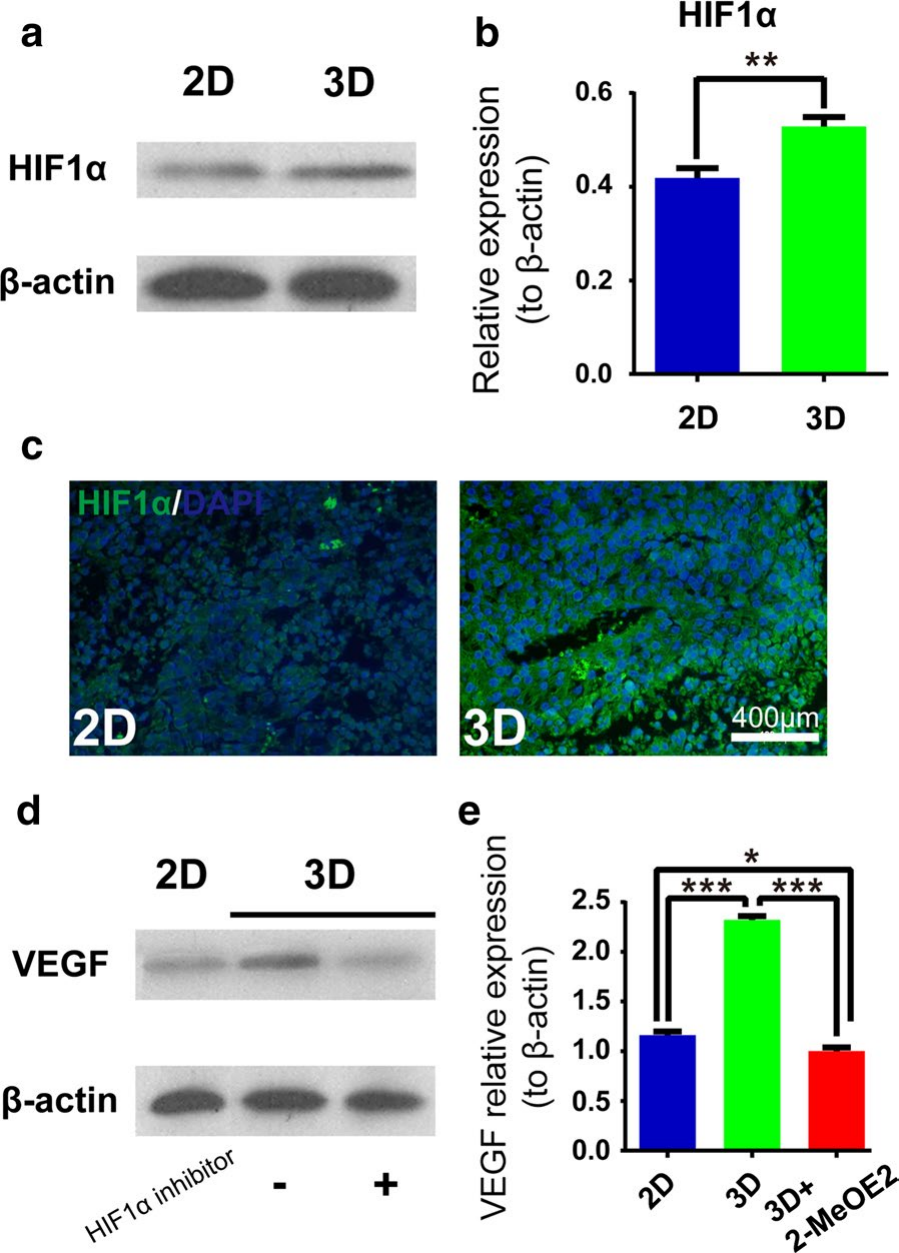
The effect of the hypoxic environment in 3D culture on the increased expression of the pro-angiogenic inducer VEGF-A in low-passage poorly-differentiated human MEC cells. **a**, **b** Western blotting of HIF1α gene expression in 2D versus 3D cultured cancer cells. 2D vs. 3D: **P < 0.01. **c** Representative microscopic images of HIF1α immunofluorescence staining in 2D versus 3D cultured cancer cells. **d**, **e** Western blotting of VEGF-A gene expression in 3D cultured cancer cells in the absence or presence of the HIF1α specific inhibitor 2-MeOE2. 2D vs. 3D: ***P < 0.001; 3D vs. 3D + 2-MeOE2: ***P < 0.001; 3D vs. 3D + 2-MeOE2: *P < 0.05. Three independent repeats, with each triplicates, were performed

Next, we examined the hypoxic environment in 3D versus 2D culture, since tumor hypoxia has been recognized as a critical environment factor for tumor angiogenesis. Either western blotting (Fig. 5a, b) or immunofluorescence (Fig. 5c) for HIF1α showed that compared with 2D-grown cancer cells, 3D-grown cancer cells showed significantly higher expression of HIF1α, a molecular indicator of the hypoxic environment (for western blotting: 2D, 0.4190 ± 0.0205; 3D, 0.5287 ± 0.0297; *P* < 0.01). This implied that cancer cells during 3D culture experienced significant hypoxic environment. Then, we assessed the effect of hypoxia on VEGF-A gene expression during 3D culture with the use of the HIF1α inhibitor 2-MeOE2. Our results showed that specific inhibition of the HIF1α activities almost completely reversed the increased expression of VEGF-A in 3D cultured cancer cells (2D, 1.1610 ± 0.0384; 3D, 2.3220 ± 0.0383; 3D + HIF1α inhibitor, 1.0010 ± 0.0386) (Fig. 5d, e). This implied that the increased expression of VEGF-A during 3D culture would be dependent on the HIF1α activities, which were induced by hypoxia in 3D culture.

## Discussion

In this study, we established a 3D culture system for low-passage poorly-differentiated human MEC cells with alginate microencapsulation. Compared with conventional 2D adhesion culture, poorly-differentiated human high-grade MEC cells, cultured in the 3D microenvironment of APA microcapsules, proliferated more actively and showed significant higher potentials to promote tumor angiogenesis. This is due to a better modeling of the tumor microenvironment, such as hypoxia, during 3D culture.

3D culture techniques, instead of the well-established 2D monolayer cell culture, have been widely employed for in vitro experiments in recent cancer studies [9, 11]. It has reached a consensus that 3D culturing offers a better platform to recapitulate in vivo characteristics of cancer cells, therefore facilitating the translation of preclinical findings into clinical applications [9, 11]. When cultured in 3D microenvironments, heterotypic tumor cell spheroids allow tumor cells and stromal cells to build cell–cell and cell-ECM interactions and their surrounding microenvironment, both of which are critical parameters in tumor development and modulate tumor responses to therapeutic interventions [15]. The multicellular tumoroids can been grown in scaffold-free engineered devices that maximize cell–cell interactions and solute transport, such as tissue engineering bioreactors and microfluidic devices [11]. However, those multicellular spheroids under those low-adherence conditions do not capture the mechanical stiffness of cell substrates in vivo, which also contributes to tumor development [14]. Alternatively, tumor aggregates can be cultured when embedded in bioactive scaffolds such as matrigel or collagen I [11, 15]. However, these bioactive scaffolds have certain limitations, such as batch-to-batch variations and the undesirable interferences on cancer cell behaviors [11, 15]. For the reasons described above, we employed cell microencapsulation using alginate hydrogel as inert cell scaffolds to model a 3D microenvironment for low-passage poorly-differentiated human high-grade MEC cells.

As one of the naturally derived anionic polysaccharides, alginate hydrogel has many advantages over bioactive scaffolds, including their inert properties for cell behaviors, biocompatible for cell microencapsulation/culture, mechanical flexibility/stability and ease of cell recovery [13–15]. Although alginate per se is typically non-adhesive to cells, most cells show the ability to grow in non-functionalized alginates [15]. In addition, with controllable diameters, porosity and stiffness, alginate hydrogel bears a structural resemblance to the ECM [14]. Originally developed for tissue engineering [16, 17], cell microencapsulation with alginate hydrogels has been recently adopted as a suitable model for 3D in vitro culture in cancer researches [11]. Previous studies showed that alginate microencapsulation enhanced cancer cell proliferation and survival, modulated cancer cell migration and invasiveness and exacerbated tumor malignancies [14, 15, 18–21]. Our results demonstrated that alginate microencapsulation increased the proangiogenic potentials of low-passage poorly-differentiated human MEC cells. In tumor microenvironment where angiogenesis inducers override its inhibitors, the angiogenic switch is almost always activated and remains on to sustain cancer neovascularization [4]. VEGF-A and TSP-1 are the well-known angiogenesis inducer and inhibitor, respectively [4]. Accordingly, our results showed that when cultured in 3D microenvironment of APA microcapsules, low-passage poorly-differentiated human MEC cells significantly increased the autocrine production of VEGF and drastically decreased the autocrine production of TSP-1.

Hypoxia, a feature of most tumors [22], has been revealed as a critical parameter for cancer development and progression, including tumor angiogenesis [4, 22]. Tumor hypoxia simultaneously increases angiogenesis inducers, such as VEGF-A, and decreases angiogenesis inhibitors, such as TSP-1, thus activating and maintaining the angiogenic switch [4, 22]. As shown, low-passage poorly-differentiated human MEC cells in APA microcapsules formed multicellular spheroids, despite possibly the lack of a complex 3D structure seen in solid tumors. However, multicellular tumor cell aggregates enabled cancer cells to experience a controlled 3D environment seen in solid tumors, where a gradient of chemical and critical biological signals, such as nutrients and oxygen, promote the assembly of “tumors tissue type” [15]. Therefore, cancer cells in 3D are under hypoxic conditions [15, 21]. In contrast, low-passage poorly-differentiated human MEC cells in 2D monolayer culture are exposed to a uniform environment with sufficient availability of nutrients and oxygen [9, 21]. This is reflected by the differential expression of HIF1α for 2D versus 3D cultured cancer cells. As shown in the present study, hypoxia in 3D and normoxia in 2D would underlie the differences of gene expression important for cancer neovascularization. Hence, in vitro 3D culture models better present tumor environments in vivo and should be appreciated in future human MEC studies.

Another advantage of our study is the use of low-passage poorly-differentiated MEC cell lines, which would be a more physiologically and clinically relevant sources of high-grade human MEC cells [9]. While some human MEC cell lines have been established [23–25], long-term in vitro maintenance leaves those high-passage cancer cell lines vulnerable to the selection of dominant cell populations and the accumulation of additional genomic and epigenomic alterations [9]. In fact, the most commonly used established cancer cell lines have been recently shown no correlation with original clinical samples [26]. This compromises the feasibility of those high-passage established cancer cell lines for in vitro testing [9]. It has been suggested that low-passage cancer cell lines, in contrast to high-passage counterparts, well reflect the biology of the original tumor, such as morphology, growth behavior, and mutational profile [27] and are therefore a versatile resources of cancer cells for preclinical in vitro studies. In fact, recent studies showed that tumorigenic low-passage cancer cell lines are more relevant to the parental cancers [10, 28, 29].

However, there are some limitations in our present study. First, we did not directly assess angiogenesis induced by low-passage poorly-differentiated MEC cells cultured in 2D and 3D environments, neither in vitro through cell co-culture systems [5, 30] nor in vivo through xenograft into nude mice. Second, it remains to identify the ideal microencapsulation protocols using alginate hydrogel for low-passage poorly-differentiated MEC cells. It has been suggested that cell microencapsulation conditions must be optimized for any new cell line to get high cell performance [31]. The candidate parameters includes variations in M/G content of sodium alginate, alginate hydrogel modifications, gelling ions (Ca^2+^, Ba^2+^), microspheres with solid versus hollow cores, and cell conditions with single cells versus spheroids [31]. This screening will boost preclinical in vitro studies of MEC using 3D cell culture models.

## Conclusion

When cultured in a 3D microenvironment by alginate microencapsulation, low-passage poorly differentiated human MEC cells, derived from human patient samples of high-grade MEC, change their proliferation behavior and show significant intensification of key molecular processes for tumor angiogenesis. This is due to a better modeling of the hypoxic tumor microenvironment during 3D culture. These results reinforce the utilization of 3D cell cultures, such as alginate microencapsulation, to mimic the tumor microenvironment in vitro for performing in vitro cancer studies more relevant to clinical conditions.

## Abbreviations

2D: two-dimensional
3D: three-dimensional
APA: alginate-PLL-alginate
bFGF: basic fibroblast growth factor
ECM: extracellular matrix
ELISA: enzyme linked immunosorbent assay
FBS: fetal bovine serum
HIF1α: hypoxia-inducible factor 1 alpha
Id-1: inhibitors of DNA binding/differentiation protein 1
MEC: mucoepidermoid carcinoma
MTT: methylthiazolyl tetrazolium
PFA: paraformaldehyde
SGC: salivary gland carcinoma
TSP-1: thrombospondin 1
VEGF-A: vascular endothelial growth factor A

## References

[1] Coca-Pelaz A, Rodrigo JP, Triantafyllou A, Hunt JL, Rinaldo A, Strojan P (2015). Salivary mucoepidermoid carcinoma revisited. Eur Arch Otorhinolaryngol.

[2] Bell D, El-Naggar AK (2013). Molecular heterogeneity in mucoepidermoid carcinoma: conceptual and practical implications. Head Neck Pathol..

[3] Wang K, McDermott JD, Schrock AB, Elvin JA, Gay L, Karam SD (2017). Comprehensive genomic profiling of salivary mucoepidermoid carcinomas reveals frequent BAP1, PIK3CA, and other actionable genomic alterations. Ann Oncol.

[4] Hanahan D, Weinberg RA (2011). Hallmarks of cancer: the next generation. Cell.

[5] Yang S, Guo LJ, Gao QH, Xuan M, Tan K, Zhang Q (2010). Derived vascular endothelial cells induced by mucoepidermoid carcinoma cells: 3-dimensional collagen matrix model. J Zhejiang Univ Sci B..

[6] Yang S, Wang XY, Guo LJ, Tang XF, Gao QH, Xuan M (2008). Correlation between the expression of thrombospondin-1 and neovascularization in mucoepidermoid carcinoma. Chin Med J (Engl).

[7] Yang S, Guo LJ, Tang XF, Tan K, Gong RG, Li A (2010). The alteration of Id-1 and TSP-1 expression in mucoepidermoid carcinoma associated with its clinical features and prognosis. Int J Oral Maxillofac Surg.

[8] Volpert OV, Pili R, Sikder HA, Nelius T, Zaichuk T, Morris C (2002). Id1 regulates angiogenesis through transcriptional repression of thrombospondin-1. Cancer Cell.

[9] Choi SY, Lin D, Gout PW, Collins CC, Xu Y, Wang Y (2014). Lessons from patient-derived xenografts for better in vitro modeling of human cancer. Adv Drug Deliv Rev.

[10] Ray S, Langan RC, Mullinax JE, Koizumi T, Xin HW, Wiegand GW (2012). Establishment of human ultra-low passage colorectal cancer cell lines using spheroids from fresh surgical specimens suitable for in vitro and in vivo studies. J Cancer..

[11] Xu X, Farach-Carson MC, Jia X (2014). Three-dimensional in vitro tumor models for cancer research and drug evaluation. Biotechnol Adv.

[12] Barnes L, Eveson JW (2005). Pathology and genetics of head and neck tumors.

[13] Sánchez P, Hernández RM, Pedraz JL, Orive G (2013). Encapsulation of cells in alginate gels. Methods Mol Biol.

[14] Xu XX, Liu C, Liu Y, Li N, Guo X, Wang SJ (2013). Encapsulated human hepatocellular carcinoma cells by alginate gel beads as an in vitro metastasis model. Exp Cell Res.

[15] Estrada MF, Rebelo SP, Davies EJ, Pinto MT, Pereira H, Santo VE (2016). Modelling the tumour microenvironment in long-term microencapsulated 3D co-cultures recapitulates phenotypic features of disease progression. Biomaterials.

[16] Lim F, Sun AM (1980). Microencapsulated islets as bioartificial endocrine pancreas. Science.

[17] Orive G, Hernández RM, Gascón AR, Calafiore R, Chang TM, De Vos P (2003). Cell encapsulation: promise and progress. Nat Med.

[18] Wang Q, Li S, Xie Y, Yu W, Xiong Y, Ma X (2006). Cytoskeletal reorganization and repolarization of hepatocarcinoma cells in APA microcapsule to mimic native tumor characteristics. Hepatol Res..

[19] Leal-Egaña A, Fritsch A, Heidebrecht F, Díaz-Cuenca A, Nowicki M, Bader A (2012). Tuning liver stiffness against tumours: an in vitro study using entrapped cells in tumour-like microcapsules. J Mech Behav Biomed Mater.

[20] Shakibaei M, Kraehe P, Popper B, Shayan P, Goel A, Buhrmann C (2015). Curcumin potentiates antitumor activity of 5-fluorouracil in a 3D alginate tumor microenvironment of colorectal cancer. BMC Cancer..

[21] Angiolini VA, Cruz CU, López ML, Simon L, Matte U (2017). Alginate-embedded HuH-7 cells increase MMP-9 and reduce OCLN expression in vitro. Cancer Cell Int..

[22] Wilson WR, Hay MP (2011). Targeting hypoxia in cancer therapy. Nat Rev Cancer.

[23] Zhang L, Li L, Wang Y, Liu Y, Li C (2014). MC3 Mucoepidermoid carcinoma cell line enriched cancer stem-like cells following chemotherapy. Oncol Lett..

[24] Wang J, Chen J, Zhang K, Zhao Y, Nör JE, Wu J (2011). TGF-β1 regulates the invasive and metastatic potential of mucoepidermoid carcinoma cells. J Oral Pathol Med.

[25] Warner KA, Adams A, Bernardi L, Nor C, Finkel KA, Zhang Z (2013). Characterization of tumorigenic cell lines from the recurrence and lymph node metastasis of a human salivary mucoepidermoid carcinoma. Oral Oncol.

[26] Gillet JP, Calcagno AM, Varma S, Marino M, Green LJ, Vora MI (2011). Redefining the relevance of established cancer cell lines to the study of mechanisms of clinical anti-cancer drug resistance. Proc Natl Acad Sci USA..

[27] Lange F, Franz B, Maletzki C, Linnebacher M, Hühns M, Jaster R (2014). Biological and molecular effects of small molecule kinase inhibitors on low-passage human colorectal cancer cell lines. Biomed Res Int.

[28] Boot A, van Eendenburg J, Crobach S, Ruano D, Speetjens F, Calame J (2016). Characterization of novel low passage primary and metastatic colorectal cancer cell lines. Oncotarget..

[29] Rowehl RA, Burke S, Bialkowska AB, Pettet DW, Rowehl L, Li E (2014). Establishment of highly tumorigenic human colorectal cancer cell line (CR4) with properties of putative cancer stem cells. PLoS ONE..

[30] Bray LJ, Binner M, Holzheu A, Friedrichs J, Freudenberg U, Hutmacher DW (2015). Multi-parametric hydrogels support 3D in vitro bioengineered microenvironment models of tumour angiogenesis. Biomaterials.

[31] Rokstad AM, Gustafsson BI, Espevik T, Bakke I, Pfragner R, Svejda B (2012). Microencapsulation of small intestinal neuroendocrine neoplasm cells for tumor model studies. Cancer Sci.

